# Adipose-derived stem cells ameliorate atopic dermatitis by suppressing the IL-17 expression of Th17 cells in an ovalbumin-induced mouse model

**DOI:** 10.1186/s13287-022-02774-7

**Published:** 2022-03-07

**Authors:** Jingyan Guan, Yibao Li, Feng Lu, Jingwei Feng

**Affiliations:** Department of Plastic and Cosmetic Surgery, Nanfang Hospital, Southern Medical University, 1838 Guangzhou North Road, Guangzhou, 510515 Guangdong People’s Republic of China

**Keywords:** Adipose-derived stem cells, Atopic dermatitis, IL-17 signaling pathway, Th17 cells, Ovalbumin, Mouse model

## Abstract

**Background:**

Mesenchymal stem cells (MSCs) have therapeutic potential for atopic dermatitis (AD) owing to their immunoregulatory effects. However, the underlying mechanisms associated with the therapeutic efficacy of MSCs on AD are diverse and related to both cell type and delivery method.

**Objectives:**

This study investigated the therapeutic effect and mechanisms of adipose-derived stem cells (ADSCs) on AD using an ovalbumin (OVA)-induced AD mouse model.

**Methods:**

AD mice were subcutaneously injected with mouse ADSCs, cortisone, or PBS, and the therapeutic effects were determined by gross and histological examinations and serum IgE levels. Additionally, qPCR, RNA-sequencing analyses of skin samples and co-culture of ADSCs and Th17 cells were conducted to explore the underlying therapeutic mechanisms.

**Results:**

ADSCs treatment attenuated the AD pathology, decreased the serum IgE levels, and decreased mast cells infiltration in the skin of the model mice. Moreover, tissue levels of IL-4R and Th17-relevant products (IL-17A, CCL20, and MMP12) were suppressed in the ADSC- and cortisone-treated groups. Genomics and bioinformatics analyses demonstrated significant enrichment of inflammation-related pathways in the downregulated genes of the ADSC- and cortisone-treated groups, specifically the IL-17 signaling pathway. Co-culture experiments revealed that ADSCs significantly suppressed the proliferation of Th17 cells and the expression of proinflammatory cytokines (IL-17A and RORγT). Furthermore, expression levels of PD-L1, TGF-β, and PGE2 were significantly upregulated in co-cultured ADSCs relative to those in monocultured ADSCs.

**Conclusion:**

ADSCs ameliorate OVA-induced AD in mice mainly by downregulating IL-17 secretion of Th17 cells.

**Supplementary Information:**

The online version contains supplementary material available at 10.1186/s13287-022-02774-7.

## Background

Atopic dermatitis (AD), also known as atopic eczema, is a chronic and recurrent skin disease that affects up to 20% of children and 10% of adults worldwide and significantly reduces the quality of life for affected individuals [1–4]. Currently, major risk factors for AD are considered to be genetic predisposition (e.g., genetic mutations in the filaggrin gene [5, 6]) and environmental factors (e.g., air pollution [7], diet [8], and stress [9]). AD pathophysiology involves a complex interaction between epidermal barrier disruption, immune dysregulation, and skin microbiome abnormality [10]. Although these dysfunctions form an inseparable vicious circle, it has been considered that dysregulation of helper T (Th) cell immune responses plays a central role in AD pathogenesis and development, especially in Th2 immune responses [11]. Recent studies have demonstrated that in addition to classical Th2, other subsets of Th cells also contribute to the AD pathogenesis (e.g., Th1, Th17, and Th22) [12, 13]. Therefore, treatments that reduce the Th-mediated immune response have been the mainstream therapeutic methods for AD [14]. These treatments include nonspecific anti-inflammatory and immunosuppressive drugs, such as corticosteroids, calcineurin inhibitors, leukotriene receptor antagonists, and antihistamines. However, these drugs only provide temporary and limited symptom relief. Additionally, some are associated with adverse effects and drug resistance following long-term treatment [15, 16]. In recent years, significant progress has been made toward increasing our understanding of AD pathogenesis, and the development of novel therapies that are safe and effective for AD has been intensely pursued.

Mesenchymal stem cells (MSCs) are a promising cell-based treatment option for AD due to their unique tissue regenerative capacity and immunomodulatory ability [17, 18]. In general, MSCs can be extracted from multiple tissues, such as bone marrow, umbilical cord, adipose tissue, and dental pulp, among others [19]. Compared with other types of MSCs, adipose-derived stem cells (ADSCs) have several advantages. In particular, adipose tissue can be readily obtained through liposuction with a small incision, and ADSCs can be isolated in large amounts via relatively simple procedures. In the previous decade, several preclinical studies reported the use of ADSCs to treat AD [20–22], with these studies demonstrating ADSC-based cytotherapy as a promising method for AD treatment according to therapeutic mechanisms mainly attributed to the paracrine effect of the administered cells. Despite the encouraging results, these studies failed to comprehensively analyze the complex pathogenesis of AD and the mechanism of interaction between MSCs and AD pathogenesis-related immune cells. Moreover, the therapeutic potential of ADSCs may vary depending on the animal model, cell type, and the routine of administration, among others [20, 23–25]. Therefore, further investigation is needed to elucidate the specific mechanisms associated with ADSC-specific treatment of AD.

This study aimed to provide insight into the pathological changes that occur in AD skin and broaden the understanding of the therapeutic effect of ADSCs on AD pathogenesis. Ovalbumin (OVA)-induced AD mice were employed as a model, with these mice subcutaneously administered with mouse ADSCs and evaluated for changes in clinical severity, histological signatures of AD, and serum immunoglobulin E (IgE) levels. Additionally, this study used high-throughput RNA-sequencing analysis to investigate the therapeutic mechanisms of ADSCs on AD and identified the interleukin (IL)-17 signaling as a possible target of ADSC-specific effects. Furthermore, the underlying mechanism of the anti-atopic effect of ADSCs was investigated by in vitro co-culture of ADSCs with Th17 cells, and the pivotal cytokines in relation to cellular interactions were also explored.

## Materials and methods

### Animals

All animal procedures were approved by the Institutional Animal Care and Use Committee of Nanfang Hospital (No. NFYY-2020-0294), and all operations were performed in accordance with the National Health and Medical Research Council (People’s Republic of China) guidelines. All surgical procedures were performed according to the aseptic principle.

### Isolation and culture of mouse ADSCs

ADSC isolation was performed as previously described [26]. Female BALB/c mice (6 weeks old, *n* = 6) were purchased from Nanfang Hospital Animal Center (Guangzhou, China). After shaving, mice were sacrificed, and subcutaneous fat was harvested. Approximately 1.5 g of fat was acquired from each mouse and stored in a sterile 50-ml centrifuge tube. Subsequently, red blood cells were removed by washing three times with phosphate-buffered saline (PBS; pH 7.4), and the isolated fat was cut into small pieces, after which it was digested with 0.2% type Ι collagenase (Sigma-Aldrich, St. Louis, MO, USA) for 45 min at 37 °C with continuous stirring. After digestion, the stromal vascular fraction (SVF) was separated from the adipose tissue by centrifugation (200*g*, 5 min), resuspended with PBS, and filtered to remove large debris. The SVF suspension then underwent an additional round of centrifugation (200*g*, 5 min), followed by resuspension in a complete growth medium comprising Dulbecco’s modified Eagle’s medium (DMEM)-low glucose (Gibco-BRL, Carlsbad Island, NY, USA) supplemented with 10% fetal bovine serum (FBS; Gibco-BRL, Carlsbad Island, NY, USA) and 1% penicillin/streptomycin (DMEM-low glucose+; Gibco-BRL, Carlsbad Island, NY, USA). The SVF suspension was then placed in T75 cell culture flasks at a density of approximately 5 × 10^5^ cells/flask (P0) and incubated at 37 °C with 5% carbon dioxide (CO_2_) and 95% humidity. The medium was changed every 3 days, and cells were passaged at 90% confluence. ADSCs within the range of passage 3 to passage 5 were used for subcutaneous injection.

### In vitro differentiation of ADSCs

To analyze cell differentiation potential, the chondrogenic, osteogenic, and adipogenic differentiations of ADSCs were induced as described previously [27]. For chondrogenic differentiation, ADSCs were cultured in a chondrogenic medium (Cyagen Biosciences, Santa Clara, CA, USA) for 4 weeks. For osteogenic differentiation, ADSCs were cultured in an osteogenic medium (Cyagen Biosciences, Santa Clare, CA, USA) for 4 weeks. For adipogenic differentiation, ADSCs were cultured in an adipogenic medium (Cyagen Biosciences, Santa Clara, CA, USA) for 2 weeks. All cells were incubated at 37 °C with 5% CO_2_ and 95% humidity. Corresponding mediums were changed every 3 days. After induction, the cells were fixed in 4% paraformaldehyde solution and assessed by Alcian Blue, Alizarin Red, and Oil Red O staining (Cyagen Biosciences, Santa Clara, CA, USA), respectively.

### Murine model of AD

Female BALB/c mice (6-weeks old, *n* = 24) were randomly divided into 4 groups (*n* = 6 per group) as follows: (1) untreated mice (normal control); (2) OVA-sensitized and PBS-treated mice (PBS group); (3) OVA-sensitized and ADSC-treated mice (ADSC group); and (4) OVA-sensitized and cortisone cream-treated mice (cortisone group). Mice were housed at 25 °C under pathogen-free conditions, with a 12-/12-h light/dark cycle and ad libitum access to food and water.

**Fig. 1:**
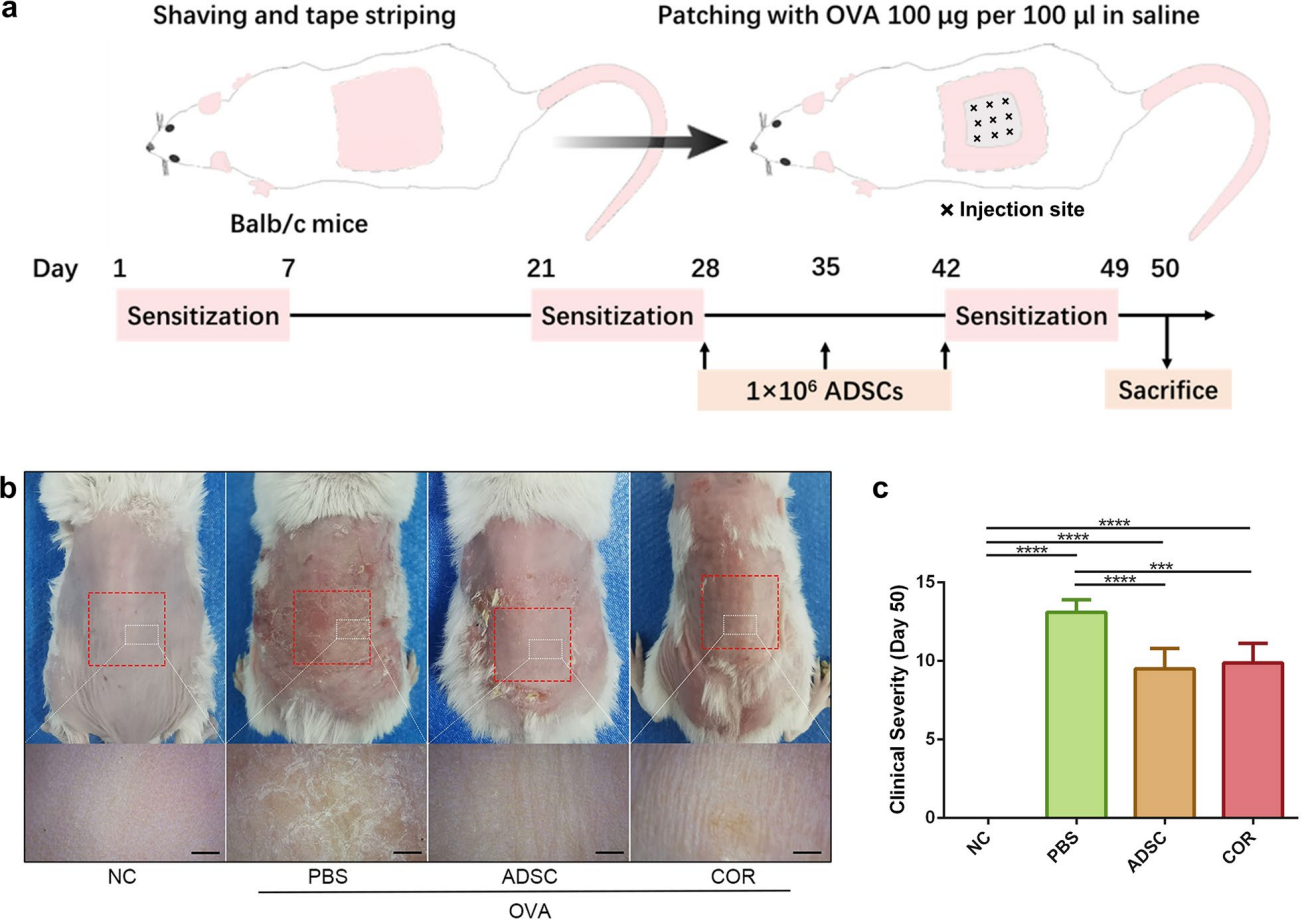
ADSCs alleviate the gross symptoms of OVA-induced AD in a murine model. **a** Schematic of OVA sensitization and ADSC application on the AD mouse model. × represents the injection site. **b** Representative macroscopic photographs and enlarged images of mice dorsal skin lesions on day 50. Red dotted boxes represent the areas covered by OVA patches. White dotted boxes represent the areas of the enlarged images. Scale bar = 1 mm. **c** Clinical severity of mice dorsal skin lesions on day 50. *n* = 6 per group. ***p < 0.001; ****p < 0.0001. AD, atopic dermatitis; OVA, ovalbumin; NC, normal control; PBS, phosphate-buffered saline; ADSC, adipose-derived stem cell; COR, cortisone

The murine model of AD was prepared as described previously [28]. Briefly, all mice (except for those in the normal control group) were anesthetized with isoflurane, and their dorsal skin was shaved, followed by applying 3 M tape (Tegaderm; Owens and Minor, Mechanicsville, VA, USA) stripped six times. Subsequently, each mouse was sensitized with a sterile patch soaked with 100 μl of 100 μg OVA (Sigma-Aldrich, St. Louis, MO, USA). The patch was placed on the dorsal skin of each mouse and changed daily for 1 week. Each mouse was exposed to a total of three 1-week sensitizations, with 2-week intervals. On days 28, 35, and 42 of OVA sensitization and challenge, 1 ml of ADSCs suspension (1 × 10^6^) was subcutaneously injected into nine sites in the area covered by the OVA patches on the dorsum of mice in the ADSC group (Fig. 1a). As a negative control, PBS group mice were subcutaneously injected with 1 ml PBS in the same area and at the same time points. In the cortisone group, 0.1 g of cortisone cream (Dinuo Pharmaceutical Co., Ltd., Hunan, China) was daubed on the dorsal skin of each mouse at the same time points and area as a positive control. All mice were sacrificed on day 50, with half of the sensitized dorsal skin fixed in 10% formalin and the other half stored at − 80 °C for further analysis.

### Measurement of clinical severity

The severity of dorsal skin lesion was assessed according to six symptoms described in a previous study [21]: erythema/hemorrhage, oozing/crust, erosion/excoriation, swelling/edema, lichenification, and dryness. Symptom scores of 0 to 3 (none to severe) were applied by two independent investigators, with the sum of these scores defined as the clinical severity (scale 0–18).

### Histologic examination

For histologic analyses, skin samples were obtained from the dorsal skins of mice in the four groups on day 50 (24 h after patch removal). Skin samples were fixed in 10% formalin for at least 24 h and then embedded in paraffin and cut into 4 μm sections for hematoxylin and eosin (H&E) staining or Toluidine Blue (TB) staining. A microscope (Olympus Corp., Tokyo, Japan) was used to obtain photomicrographs (magnification; × 200), and 10 H&E-stained regions for each group were selected randomly for epidermal thickness measurement using the ImageJ software (NIH, Bethesda, MD, USA). Infiltrated mast cells were counted in 10 random TB-stained regions for each group.

### Measurement of serum IgE levels

On day 50, whole blood samples of mice were collected by removing the eyeballs after anesthesia. After clotting at 25 °C for 30 min, the blood samples were centrifuged at 1000 *g* for 10 min at 4 °C and serum was collected and preserved at − 80 °C until use. Serum IgE was detected using a mouse IgE enzyme-linked immunosorbent assay kit (Invitrogen, Carlsbad, CA, USA) according to the manufacturer instructions.

### RNA-sequencing analysis

Total RNA was extracted from dorsal skin samples of the 4 groups (*n* = 3 per group) using TRIzol reagent (Invitrogen, Carlsbad, CA, USA) according to the manufacturer’s instructions. RNA quantification was performed using Quant-IT RiboGreen (Invitrogen, Carlsbad, CA, USA) and RNA quality was assessed using the Bioanalyzer 2100 system (Agilent Technologies, Santa Clara, CA, USA). After quality inspection, mRNAs were purified, fragmented, and converted to first-strand complementary DNA (cDNA) via reverse transcription. The first-strand cDNA was subsequently converted to the second strand, followed by use of the AMPure XP system (Beckman Coulter, Miami, FL, USA) to purify the library fragments and screen 370- to 420-bp cDNA fragments. After library construction, polymerase chain reaction (PCR) was performed, with the products purified and verified using the AMPure XP (Beckman Coulter, Miami, FL, USA) and Agilent Bioanalyzer 2100 systems, respectively. A cluster of the index-coded samples was generated using a TruSeq PE cluster kit (v3-cBot-HS; Illumina, San Diego, CA, USA) on a cBot cluster generation system according to the manufacturer instructions. The Illumina Hiseq 3000 (Illumina, San Diego, CA, USA) was used to sequence the prepared libraries in a 150-bp paired-end reads to generate raw reads. Using in-house Perl scripts, raw reads in the fastq format were processed into clean reads and then aligned to the reference genome sequence of Mus musculus (mm10) using Hisat2 (v2.0.5). The read numbers of the genes in each group were calculated using FeatureCounts (v1.5.0-p3). R software was used for quantile normalization and subsequent data processing, and differentially expressed genes (DEGs) between each group were identified using the DESeq2 R package (v1.20.0) with a significance threshold of |log2 fold change (FC)| ≥ 1 and an adjusted *p* (padj) ≤ 0.05. The ClusterProfiler R software package was used for Gene Ontology (GO) enrichment analysis and Kyoto Encyclopedia of Gene and Genome (KEGG) enrichment analysis. GO terms and KEGG pathways with a padj ≤ 0.05 were considered significantly enriched.

### Isolation of naïve mouse CD4+ T cells and in vitro differentiation into Th17 cells

The isolation of naïve mouse CD4+ T cells was performed as described previously [29]. Briefly, the spleens and lymph nodes of BALB/c female mice (5–10-weeks old) were obtained under aseptic conditions. These two tissues were then placed in PBS supplemented with 1% penicillin/streptomycin (PBS+), followed by grinding into a suspension using two slides. After filtration through a 120 μm pore size nylon mesh (Corning Costar, Cambridge, MA, USA), the suspension was replenished using PBS+ and centrifuged at 475*g* and 4 °C for 5 min. The lymph node cell pellet was resuspended with 2 ml PBS+, and the splenic cell pellet was resuspended with ammonium–chloride–potassium lysis buffer (1 ml per spleen) for 1 min to lyse red blood cells. Then, 10 ml of PBS+ was added and centrifugated at 475*g* and 4 °C for 5 min. The splenic cell pellet was resuspended again with 10 ml PBS+ and mixed with the lymph node cell suspension. After a final round of centrifugation at 475*g* and 4 °C for 5 min, the combined cell pellet was subjected to CD4 enrichment using T cell enrichment columns (R&D Systems, Minneapolis, MN, USA). Mouse CD4+ T cell isolation kits (Miltenyi Biotec GmbH, Bergisch Gladbach, Germany) were used to purify naive CD4+ T cells through negative selection according to manufacturer instructions.

For in vitro differentiation, naive CD4+ T cells were placed in a 6-well plate coated with anti-CD3 and anti-CD28 (2 μg/ml) antibodies. The cells were cultured in Roswell Park Memorial Institute (RPMI)-1640 (Gibco-BRL, Carlsbad Island, NY, USA) supplemented with 10% FBS, 2 mM L-glutamine, 1% penicillin/streptomycin (RPMI-1640+), and Th17 polarizing cytokines [10 ng/ml transforming growth factor-beta (TGF-β), 20 ng/ml IL-6, 10 μg/ml anti-IL-4 antibody, and 10 μg/ml anti-interferon-gamma (IFN-γ) antibody]. The cells were incubated at 37 °C with 5% CO_2_ and 95% humidity for 4 to 5 days. On day 3, cells were removed to uncoated new wells and cultured in RPMI-1640+ supplemented with 10 ng/ml IL-2 in order to enhance proliferation. All cytokines and antibodies applied for differentiation of CD4+ T cells were purchased from BD Biosciences (San Diego, CA, USA).

### Co-culture of ADSCs and Th17 cells

The co-culture experiment comprised three groups: direct co-culture of ADSCs and Th17 cells (experimental group) and monoculture of Th17 cells or ADSCs (two control groups). For the co-culture group, 1 × 10^5^ ADSCs were placed into a 6-well plate and cultured overnight in 2 ml DMEM-low glucose+ until cells became adherent. The medium was then changed to 5 ml RPMI-1640+, and Th17 cells (2 × 10^6^ cells/well) were added to obtain a ratio of 20:1 with the ADSCs. For the monoculture of Th17 cells, an equal number of Th17 cells were added to a blank well and cultured with 5 ml RPMI-1640+. For the monoculture of ADSCs, 1 × 10^5^ ADSCs were placed into another blank well and cultured with 5 ml DMEM-low glucose+. The ADSCs and Th17 cells of each group were collected after 72 h of culture for further experiments.

### Quantitative real-time PCR (qPCR)

The total RNA was extracted from the skin samples and cell samples with Trizol Reagent (Invitrogen, Carlsbad, CA, USA), followed by reverse transcription into cDNA using a reverse transcription kit (DBI Bioscience, Hennigsdorf, Germany) according to the manufacturer instructions. FastStart Universal SYBR Green Master (Cat No. 04913850001; Roche, Basel, Switzerland) was used to perform qPCR. The skin samples were tested for the following genes: IL-13, IL-4, IL-4 receptor (IL-4R), IL-17A, C–C motif chemokine ligand 20 (CCL20), matrix metallopeptidase 12 (MMP12), IFN-γ, and tumor necrosis factor-alpha (TNF-α). Th17 cell samples were tested for IL-17A, IL-17F, and RAR-related orphan receptor gamma T (RORγT) and ADSC samples were tested for programed death ligand 1 (PD-L1), TGF-β, and prostaglandin E2 (PGE2). The primer–probe sequences for the genes are shown in Additional file 1: Table S1. Relative expression was determined using the 2^−ΔΔCt^ method, and β-actin was used as an endogenous reference gene.

### Statistical analysis

For RNA-sequencing analysis, statistical analysis was implemented using R software (www.r-project.org), and R packages were acquired on Bioconductor (www.bioconductor.org). DEGs between each group were identified using Student’s *t* test, with significant differences in relative expression levels determined according to a |log_2_FC|≥ 1 and padj ≤ 0.05. Significantly enriched GO terms and KEGG pathways were identified using the ClusterProfiler R package with a padj value was calculated using right-sided hypergeometric tests (padj ≤ 0.05 was considered statistically significant). For the other experiments, data were presented as the mean ± standard deviation (SD) and analyzed using SPSS (version 21.0; IBM Corp., Armonk, N.Y., USA). One-way analysis of variance, followed by the Tukey’s multiple comparison test was utilized for multiple groups. An unpaired Student’s *t* tests was used to compare data between two groups. A *p* ≤ 0.05 was considered statistically significant.

## Results

### ADSCs exhibit multipotency

The extracted ADSCs from passage 3 to 5 were subjected to chondrogenic, osteogenic, and adipogenic differentiation in vitro to test their multi-lineage differentiation potential. Cartilage glycosaminoglycans, calcium phosphates, and lipid drops produced by chondrocytes, osteocytes, and adipocytes were successfully detected by Alcian Blue (Additional file 2: Fig. S1a, red arrows), Alizarin Red (Additional file 2: Fig. S1b, yellow arrows), and Oil Red O (Additional file 2: Fig. S1c, white arrows) staining, respectively. These results indicated that the extracted ADSCs possessed multi-lineage differentiation potency.

### ADSCs ameliorate AD symptoms in an OVA-induced mouse model

The therapeutic effect of ADSCs on AD was evaluated in an AD mouse model. On day 50, serious erythema and dryness, accompanied by lichenification and excoriation, were observed on the dorsal skins of PBS-treated mice; however, skin lesions were significantly improved (only slight skin redness) in the OVA-sensitized area of the mice in the ADSC- and cortisone-treated groups (Fig. 1b). Similarly, the clinical severity of the dorsal skin lesions was significantly lower in groups treated with ADSCs (9.500 ± 1.309, *p* < 0.0001) and cortisone (9.875 ± 1.246, *p* < 0.001) on day 50 as compared with that in mice treated with PBS (13.11 ± 0.7817) (Fig. 1c).

### ADSCs suppress epidermal thickening, mast cells infiltration, and serum IgE levels in an OVA-induced mouse model

**Fig. 2:**
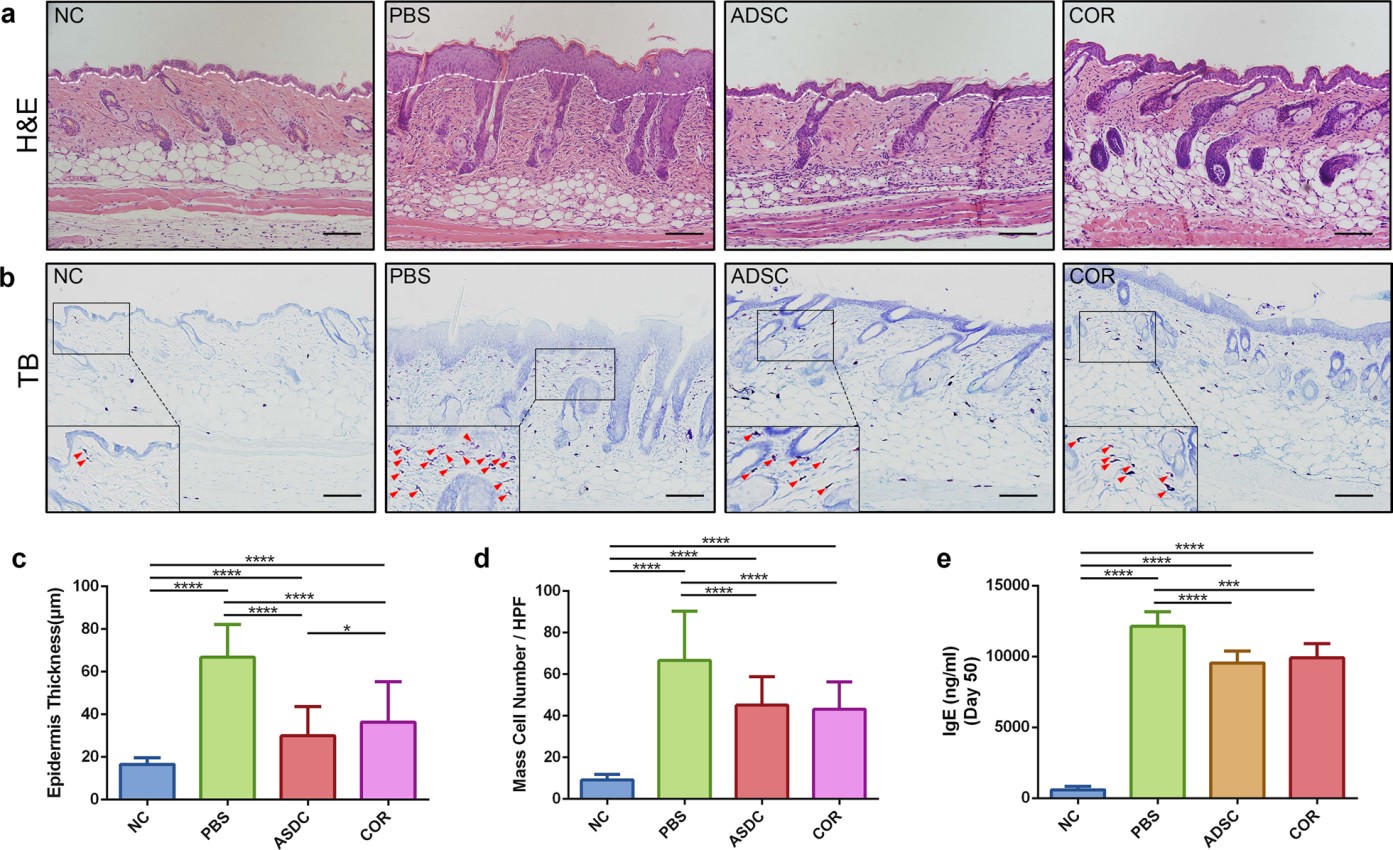
ADSCs improve histological features of lesion sites and decrease serum IgE levels in mice with OVA-induced AD. **a** Representative H&E staining photomicrographs of dorsal skin samples from the four groups. The epidermis and dermis are distinguished by white dotted lines, with the parts above the white dotted line representing the epidermal layer. Scale bar = 100 μm. **b** Representative TB staining photomicrographs of dorsal skin samples from the four groups. Red arrows show infiltrated mast cells. Scale bar = 100 μm. **c** Measurement of epidermal thickness in skin samples; *n* = 10 per group. **d** Measurement of infiltrated mast cells in skin samples; *n* = 10 per group. **e** Serum IgE level on day 50; *n* = 6 per group. *p < 0.05; ****p < 0.0001. Scale bar = 100 μm. NC, normal control; PBS, phosphate-buffered saline; ADSC, adipose-derived stem cell; COR, cortisone; IgE, immunoglobulin E; AD, atopic dermatitis; OVA, ovalbumin; TB, Toluidine Blue

To determine the impact of ADSCs on the structure of AD skin, H&E staining was performed to evaluate changes in epidermal thickness. H&E staining showed that the epidermis of the dorsal skin in PBS-treated mice exhibited remarkable thickening (66.80 ± 15.44 μm, *p* < 0.0001) as compared with that in normal control (16.51 ± 3.161 μm), whereas epidermal thickness was significantly lower in the ADSC-treated mice (29.97 ± 13.68 μm, *p* < 0.0001) and the cortisone-treated group (36.39 ± 18.89 μm, *p* < 0.0001) relative to the PBS-treated group. Additionally, epidermal thicknesses in the ADSC group was slightly lower than that in the cortisone group (*p* = 0.0081), with no statistical difference relative to the normal control (Fig. 2a, c). Toluidine Blue staining is a useful method for identifying mast cells. Numerous bluish purple-stained mast cells were observed in the skin of the PBS-treated mice, whereas fewer infiltrated mast cells were found in the skin of the ADSC- and cortisone-treated mice (Fig. 2b, red arrow heads). Semi-quantification analysis showed that the numbers of infiltrated mast cells in the skin of both ADSC- (45.11 ± 13.76, *p* < 0.0001) and cortisone-treated mice (43.09 ± 13.30, *p* < 0.0001) were significantly lower than those in the PBS-treated mice (66.60 ± 23.77) (Fig. 2d). In parallel, a significant increase in the serum IgE levels were observed in PBS-treated mice (12,127 ± 421.3 ng/ml, *p* < 0.0001) as compared with those in the normal control (598.8 ± 245.5 ng/ml) on day 50. By contrast, serum IgE levels were reduced in mice that received the ADSCs (9547 ± 832.1 ng/ml, *p* < 0.0001) or cortisone treatment (9903 ± 1014 ng/ml, *p* < 0.001) relative to those in the PBS group (Fig. 2e). These results indicated that ADSCs partially ameliorated the histological signatures and immunogenic markers of AD.

### ADSCs decrease the expression of IL-4R and Th17-associated factors in AD skin

**Fig. 3:**
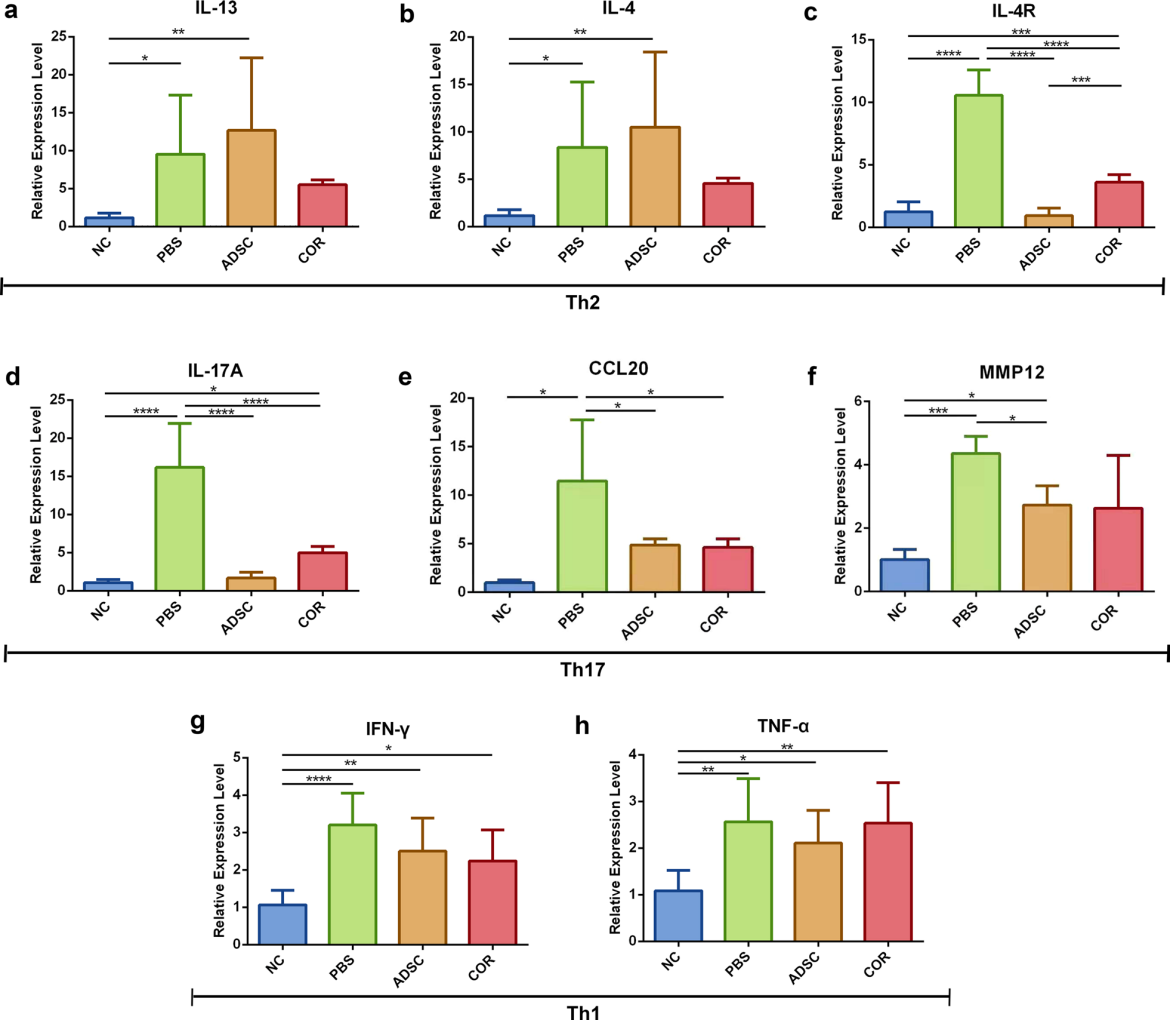
Effect of ADSCs on gene expression level of proinflammatory cytokines in OVA-induced AD mice. Relative gene expression levels of **a** IL-13, **b** IL-4, **c** IL-4R, **d** IL-17A, **e** CCL20, **f** MMP12, **g** IFN-γ and **h** TNF-α in the skin lesions measured by qPCR. *n* = 6 per group. *p < 0.05; **p < 0.01; ***p < 0.001; ****p < 0.0001. NC, normal control; PBS, phosphate-buffered saline; ADSC, adipose-derived stem cell; AD, atopic dermatitis; OVA, ovalbumin; qPCR, quantitative real-time polymerase chain reaction; COR, cortisone; IL, interleukin; IL-4R, IL-4 receptor; CCL20, C–C motif chemokine ligand 20; MMP12, matrix metallopeptidase 12; IFN-γ, interferon-gamma; TNF-α, tumor necrosis factor-alpha

Because AD pathogenesis involves dysregulation of multiple types of Th cells, qPCR was performed to detect the expression of inflammatory cytokines associated with Th2 (IL-13, IL-4, and IL-4R), Th17 (IL-17A, CCL20, and MMP12), and Th1 (TNF-α and IFN-γ) cells. The expression of the aforementioned cytokines were found markedly upregulated in the PBS group relative to that in the normal control (IL-13, *p* < 0.05; IL-4, *p* < 0.05; IL-4R, *p* < 0.0001; IL-17A, *p* < 0.0001; CCL20, *p* < 0.005; MMP12, *p* < 0.001; IFN-γ, *p* < 0.0001; and TNF-α, *p* < 0.01) (Fig. 3a–h). Compared with the PBS group, the expression of IL-4R and Th17-associated factors was significantly downregulated in the mice that received the ADSCs treatment (IL4-R, *p* < 0.0001; IL-17A, *p* < 0.0001; CCL20, *p* < 0.05; and MMP12, *p* < 0.05) (Fig. 3c–f) and cortisone treatment (IL-4R, *p* < 0.0001; IL-17A, *p* < 0.0001; and CCL20, *p* < 0.05) (Fig. 3c–e). Additionally, IFN-γ (Fig. 3g) and TNF-α (Fig. 3h) expression was slightly decreased in the ADSC- and cortisone-treated groups relative to the PBS-treated group, although the differences were not statistically significant. The expression of IL-13 (Fig. 3a) and IL-4 (Fig. 3b) was slightly increased in the ADSC-treated group as compared with the PBS-treated group, but there was no statistical significance (Fig. 3a, b). These results suggested that ADSCs treatment inhibited the expression of AD-related inflammatory factors.

### ADSCs normalize elevation of the IL-17 signaling pathway in OVA-induced AD skin lesions

**Fig. 4:**
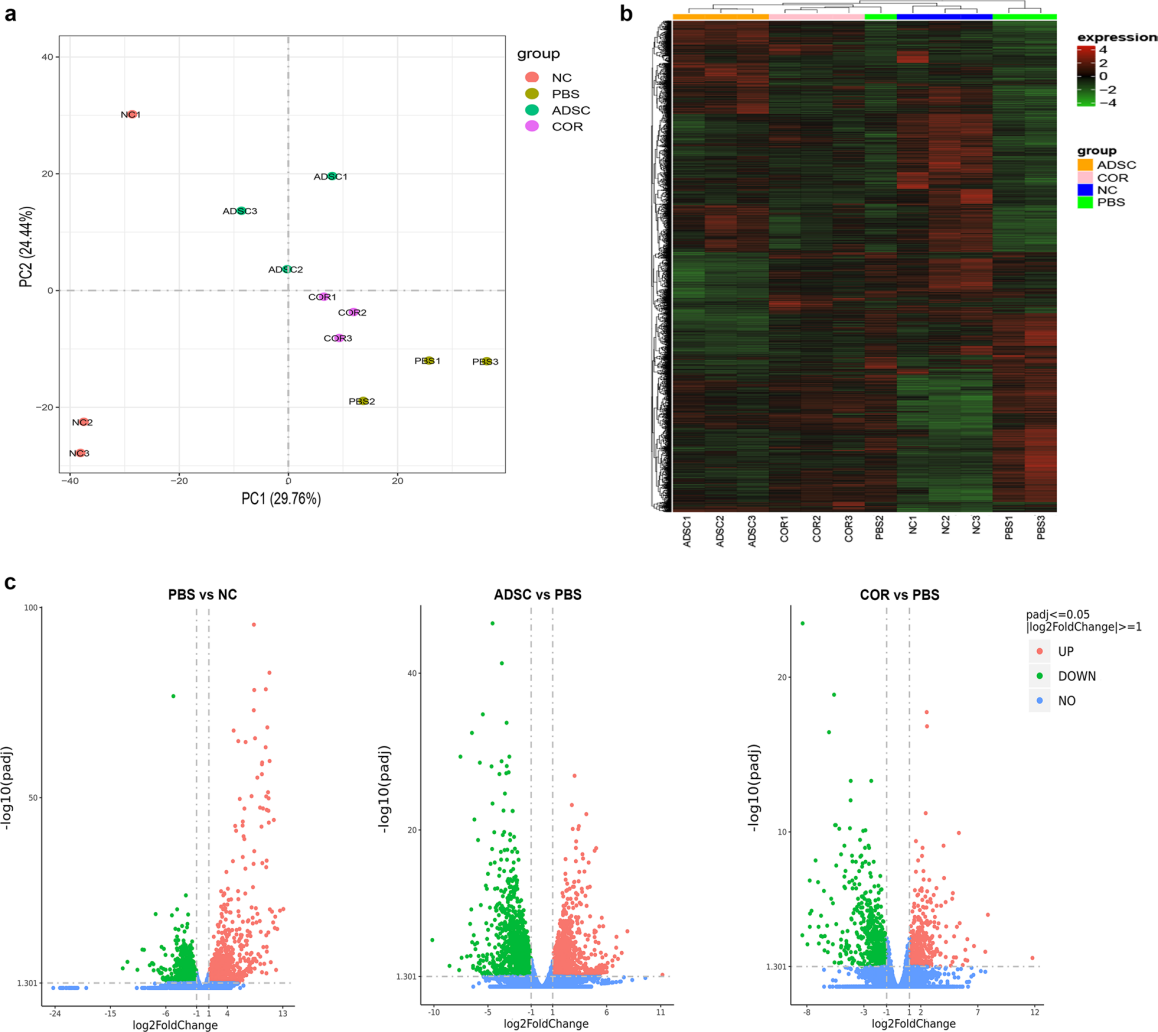
RNA sequencing of mice dorsal skin samples. **a** PCA of dorsal skin samples in the NC, PBS, ADSC, and COR groups. **b** Heat maps of DEGs between the NC, PBS, ADSC, and COR groups. **c** Volcano plots of DEGs between the PBS and NC groups (left), the ADSC and PBS groups (middle), and the COR and PBS groups (right); *n* = 3 per group. NC, normal control; PBS, phosphate-buffered saline; ADSC, adipose-derived stem cell; COR, cortisone; PCA, principal component analysis; padj, adjusted *p* value

To determine the mechanisms by which ADSCs improve AD symptoms, skin lesions from the normal control, PBS, ADSC, and cortisone groups were analyzed using high-throughput RNA-sequencing analysis. Principal component analysis (PCA) showed that the ADSC group was clearly separated from the normal control, PBS, and cortisone groups (Fig. 4a, green circles), suggesting that ADSCs regulate a different gene expression program in AD restoration. According to the established thresholds, 4771 DEGs were identified from the skin specimens of the four groups (Fig. 4b), with 2965 DEGs between the PBS and normal control groups, 2641 DEGs between the ADSC and PBS groups, 933 DEGs between the cortisone and PBS groups, and 1204 DEGs between the ADSC and cortisone groups (Fig. 4c).

**Fig. 5:**
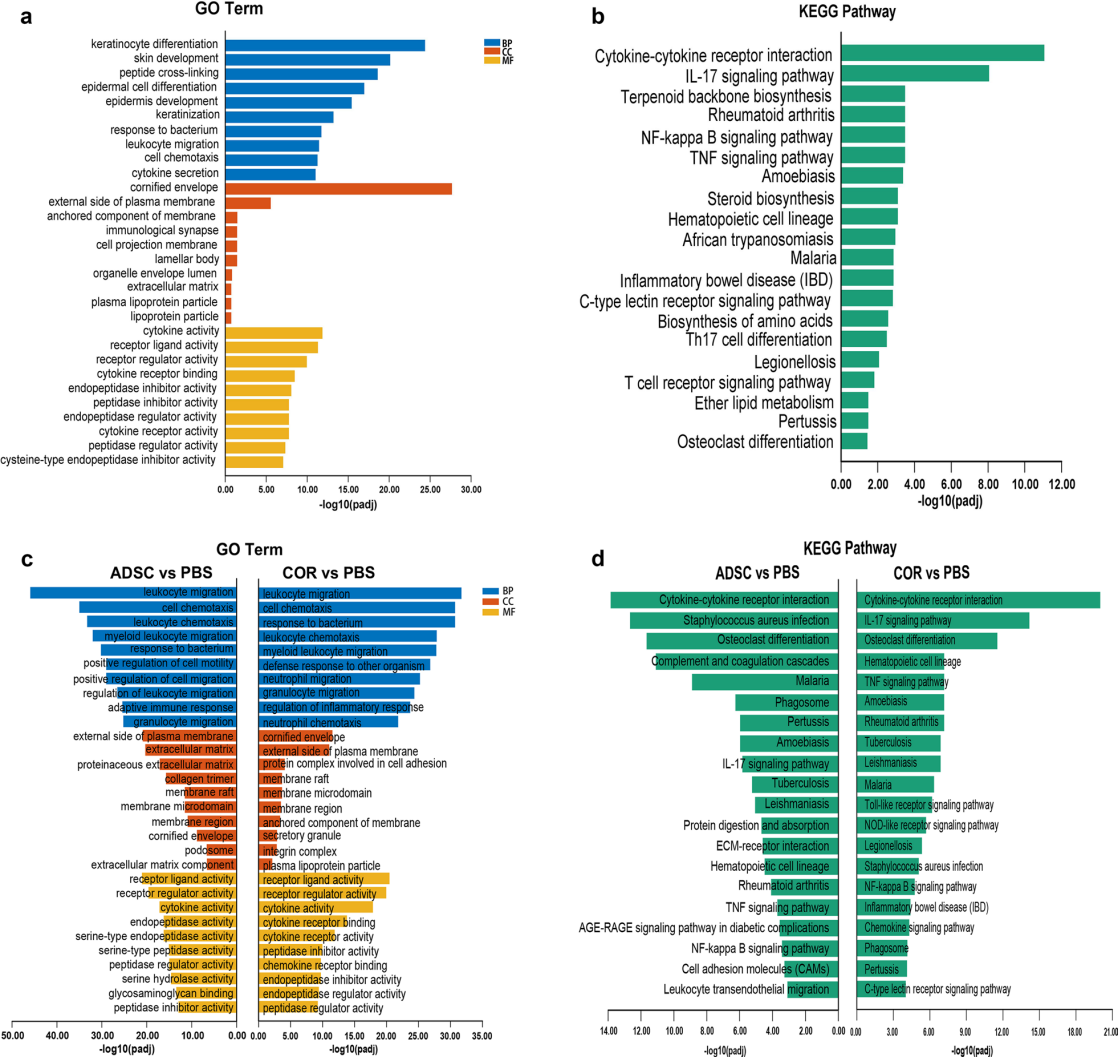
GO and KEGG analyses based on DEGs between the NC, PBS, ADSC, and COR groups. **a** Top 10 GO terms for BP, CC, and MF of the upregulated DEGs of PBS versus the NC group. **b** Top 20 enriched KEGG signaling pathways of the upregulated DEGs of PBS versus the NC group. **c** Top 10 GO terms for BP, CC, and MF of the downregulated DEGs of the ADSC versus PBS and COR versus PBS groups. **d** Top 20 enriched KEGG pathways of the downregulated DEGs of the ADSC versus PBS and COR versus PBS groups; *n* = 3 per group. NC, normal control; PBS, phosphate-buffered saline; ADSC, adipose-derived stem cell; COR, cortisone; BP, biological process; CC, cellular component; MF, molecular function; padj, adjusted *p*-value; GO, Gene Ontology; KEGG, Kyoto Encyclopedia of Gene and Genome; DEGs, differentially expressed genes

To determine the specific biological processes and signaling pathways involved in the repair of AD by ADSCs, GO and KEGG analyses were performed on the upregulated DEGs between the PBS and normal control groups. GO results showed that the top 10 terms for biological process (BP), cellular component (CC), and molecular function (MF) were mostly related to epidermis hyperplasia, leukocyte migration, and cytokine-related activities (Fig. 5a). Compared with the normal control group, KEGG analysis showed a significant change in the IL-17 signaling pathway in the PBS group, suggesting that Th17 cells were activated after OVA sensitization (Fig. 5b).

Subsequently, GO and KEGG analyses were conducted on the downregulated DEGs between the ADSC and PBS groups and between the cortisone and PBS groups. GO results showed that ADSCs and cortisone treatments downregulated the expression of genes mainly involved in leukocyte activities, bacterium response, and cytokine-related activities (Fig. 5c), indicating the alleviation of AD-related inflammatory responses after treatment. KEGG analysis showed a significant change in the IL-17 signaling pathway between the ADSC and PBS groups (ranked ninth) and between the cortisone and PBS groups (ranked second) (Fig. 5d), suggesting that ADSCs effectively suppress activation of Th17 cells, similar to cortisone treatment.

### ADSCs suppress the proliferation and activation of Th17 cells in vitro

**Fig. 6:**
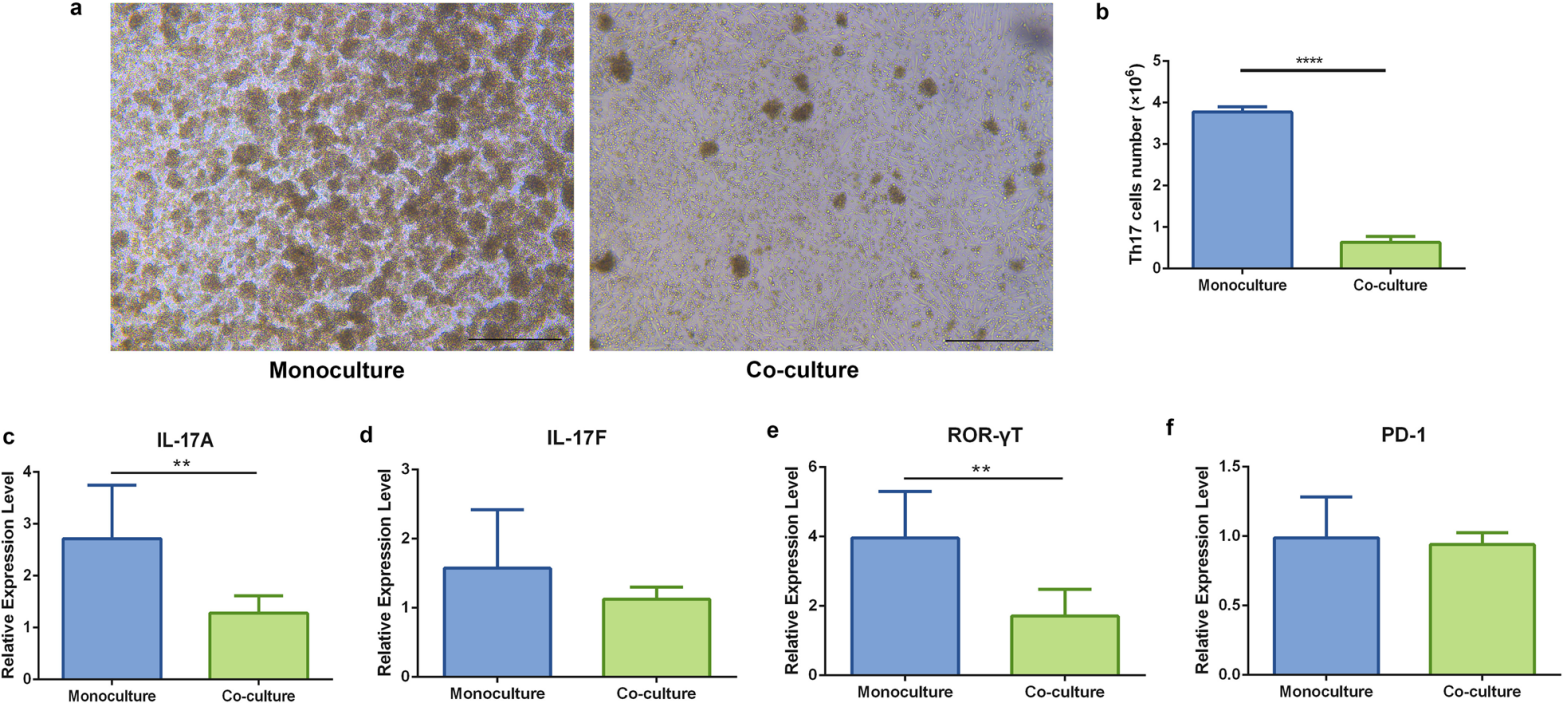
Effect of ADSCs on proliferation and activation of Th17 cells. **a** Representative photomicrographs of the monocultured (left) and co-cultured (right) Th17 cells after 72 h of incubation. Scale bar = 500 μm. **b** Cell count of the monocultured and co-cultured Th17 cells after 72 h of incubation. Relative gene expression levels of **c** IL-17A, **d** IL-17F, and **e** RORγT in the monocultured and co-cultured Th17 cells determined by qPCR. *n* = 5 per group. *p < 0.05; **p < 0.01; ****p < 0.0001. ADSCs, Adipose-derived stem cells; interleukin, IL; RORγT, RAR-related orphan receptor gamma T; qPCR, quantitative real-time polymerase chain reaction

To identify the mechanism associated with ADSC-mediated inhibition of Th17 cell activation, a direct co-culture of ADSCs and Th17 cells was conducted. After 72 h of incubation, fewer T cell receptor-activated T cell clusters, indicators of T cell proliferation, were observed in the co-culture group relative to those observed in monocultured Th17 cells (Fig. 6a). Additionally, monocultured Th17 cells proliferated to 3.774 ± 0.1297 × 10^6^ in 72 h, whereas only 0.6310 ± 0.1465 × 10^6^ Th17 cells were counted in the co-culture group, which was also fewer than the initial cell number (2 × 10^6^) (*p* < 0.0001, Fig. 6b). Correspondingly, the expression levels of IL-17A and RORγT, the master transcription factor associated with Th17 cell differentiation, were significantly reduced under the co-culture conditions (IL-17A, *p* < 0.01; and RORγT, *p* < 0.01) (Fig. 6c, e). Furthermore, IL-17F expression was reduced in co-cultured Th17 cells, although there was no significant difference relative to that in monocultured Th17 cells (Fig. 6d). These results indicated that ADSCs treatment suppressed the proliferation and activation of Th17 cells.

**Fig. 7:**
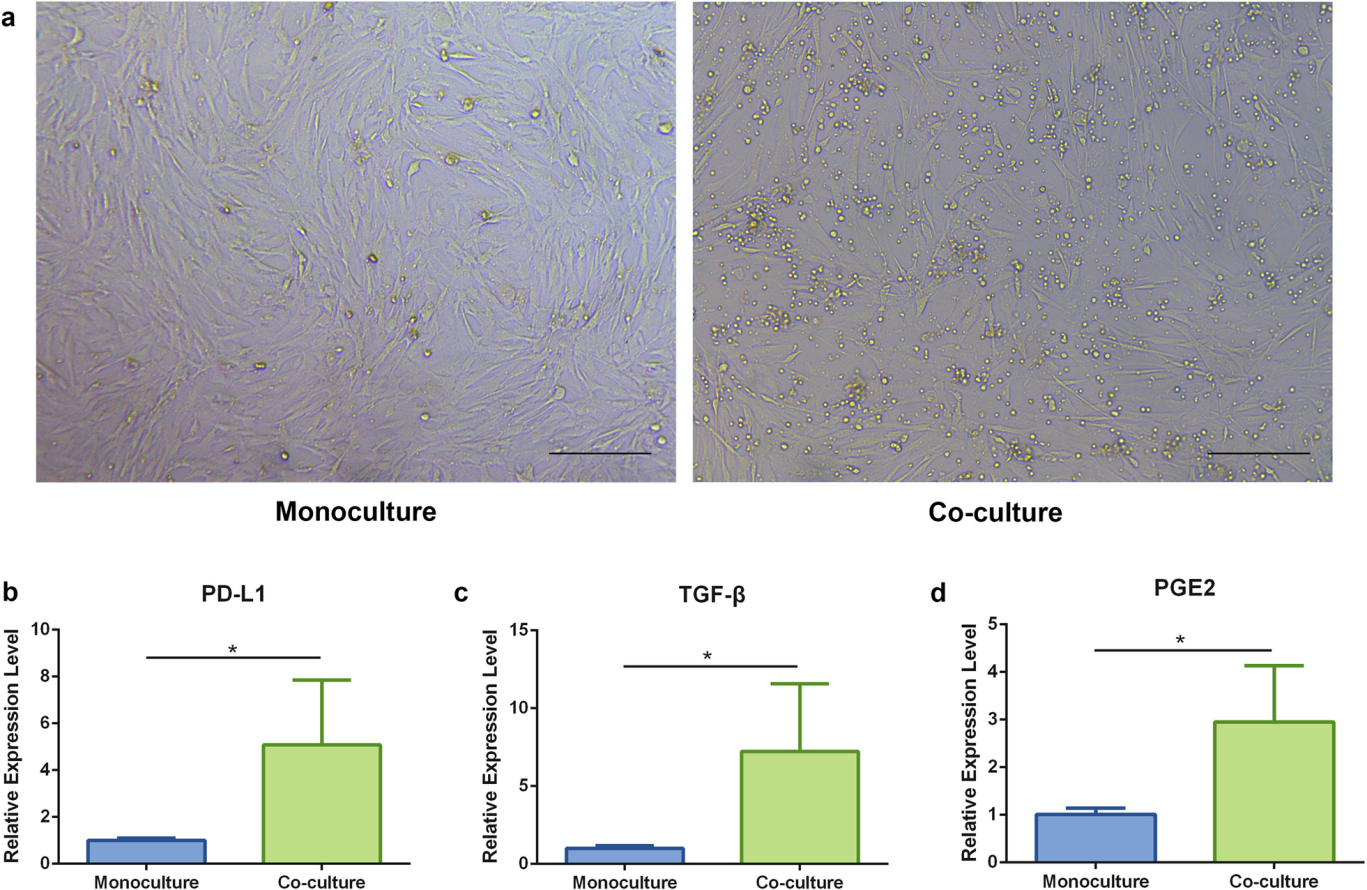
Gene expression level of PD-L1, TGF-β, and PGE2 in ADSCs. **a** Representative photomicrographs of the monocultured (left) and co-cultured (right) ADSCs after 72 h of incubation. Scale bar = 200 μm. Relative gene expression levels of **b** PD-L1, **c** TGF-β, and **d** PGE2 in the monocultured and co-cultured Th17 cells determined by qPCR. *n* = 5 per group. *p < 0.05. ADSCs, Adipose-derived stem cells; PD-L1, programed death ligand 1; TGF-β, transforming growth factor-beta; PGE2, prostaglandin E2; qPCR, quantitative real-time polymerase chain reaction

As shown in Fig. 7a, no obvious morphological differences were observed between co-cultured and monocultured ADSCs. Furthermore, the expression of PD-L1, TGF-β, and PGE2, which reportedly participate in MSC-specific immunomodulatory effects [30, 31], was significantly upregulated in the co-cultured ADSCs as compared with their expression in monocultured ADSCs (PD-L1, *p* < 0.05; TGF-β, *p* < 0.05; PGE2, *p* < 0.05) (Fig. 7b–d).

## Discussion

In this study, subcutaneous administration of ADSCs was found ameliorated OVA-induced murine AD, potentially through downregulation of IL-17 secretion by Th17 cells. The delivery methods of MSCs in AD models mainly include subcutaneous and intravenous injections [22, 32]. Although a previous study reported that ADSCs have homing effects to the injured tissues after intravenous injection [33], another report indicated that this administration route would lead to low viability of stem cells because of pulmonary interception [34]. Additionally, another report demonstrated that local injection of ADSCs resulted in higher viability than intravenous injection [24]. Therefore, in the present study, ADSCs were administrated directly to lesion sites by subcutaneous injection and successful relief of AD symptoms was observed.

IgE–mast cells interactions are known to play a pivotal role in many allergic and inflammatory responses [35, 36]. Mast cells activated by IgE degranulate to release various proinflammatory mediators and contribute to the persistence of AD symptoms [37, 38]. Ando et al. [39] reported that inhibition of mast cell activities leads to the amelioration of AD, whereas increased mast cell activities aggravate AD symptoms. In the present study, significantly lower numbers of infiltrated mast cells and serum IgE levels were observed following ADSCs treatment, suggesting an inhibitory effect of the ADSCs on mast cells recruitment and local allergic responses. This inhibitory effect of MSCs on mast cells was also confirmed in a previous study using dinitrochlorobenzene-induced AD BALB/c mice treated with human-derived ADSCs and in OVA-induced BALB/c mice treated with superoxide dismutase 3-transduced MSCs [40]. A recent study reported that the number of mast cells and serum IgE levels were nearly threefold higher in the control group relative to those in AD model mice treated with human umbilical cord blood-derived MSCs [41]. Additionally, Park et al. [21] reported a nearly 3.2-fold decrease in the number of mast cells in skin samples from AD mice treated with autologous ADSCs relative to that in control mice. In the present study, the number of mast cells and serum IgE levels were observed ~ 1.5- and 1.3-fold higher in PBS-treated AD mice, respectively, relative to those in ADSC-treated mice. The authors speculated that the differences in therapeutic effectiveness and mast cell populations in response to stem cells treatment might be attributed to the diversity in cell lines and animal models.

As a mediator for IL-4 and IL-13 acting on downstream inflammatory cells, IL-4R complex participate in multiple signaling pathways that related to AD immune dysregulation [42, 43].Yun et al. [44] reported treating 2,4-dinitrochlorobenzene (DNCB)-induced AD mice with echinochrome A (an anti-inflammatory and anti-oxidative drug), revealing that echinochrome A appeared to suppress the expression and secretion of the proinflammatory cytokine IL-4 and IL-13. A recent study showed that the clinical features of experimental atopic dermatitis are driven by IL-4 and IL-13 signaling via IL-4R and highlighted that IL-4R might serve as a potent therapeutic target for the treatment of AD and other allergic diseases [45]. In the present study, ADSCs treatment downregulated the expression of IL-4R rather than IL-4 and IL-13, indicating that ADSCs might inhibit Th2-mediated inflammation by suppressing IL-4R-specific activity in various downstream inflammatory cells but not by directly decreasing the production of Th2-related cytokines. However, the underlying mechanisms remain to be clarified.

Recent studies indicate that Th17 cells are profoundly involved in the pathogenesis of certain skin disorders and autoimmune diseases [46–48]. Consistent with previous studies [49–51], the present study identified increases in the expression of the general inflammation marker MMP12 and the Th17 marker CCL20 in AD skin lesions, whereas ADSC treatment reversed these changes. Cytokines secreted by Th17 cells were reported to stimulate CCL20 expression by keratinocytes, which in turn recruits more Th17 cells and creates a positive feedback loop to maintain the inflammatory state in skin lesions [52, 53]. Notably, the observed reduction in CCL20 expression in ADSC-treated AD skin indicated attenuation of the feedback loop, possibly through inhibition of Th17-related cytokine production by ADSCs. IL-17, a key cytokine for Th17 cells, reportedly participates in allergen-specific immune responses [54, 55], exhibits increased expression in skin trauma and skin barrier dysfunction-associated diseases [56], regulates epidermal cell proliferation conditions and skin thickening [57]. Given the proinflammatory properties, IL-17 was subsequently investigated. A previous study reported that application of the anti-inflammatory agent artesunate significantly decreased the expression of IL-17 and improved the histopathological symptoms in DNCB-induced AD mice [58]. Studies on the interaction between MSCs and Th17 cells showed that MSCs and their exosomes treatment can significantly reduce the percentage of Th17 cells and the expression of IL-17 in experimental autoimmune uveitis [59] and Sjögren’s syndrome [60] models. The present results revealed that the mRNA expression of IL-17 in skin samples was effectively inhibited in ADSC-treated mice relative to that in PBS-treated mice. Moreover, RNA-sequencing analysis identified a remarkable downregulation of IL-17 signaling pathways in ADSC-treated mice, indicating that the therapeutic effect of ADSCs on AD may be attributable to downregulating IL-17 secretion by Th17 cells. A recent study demonstrated that Th17-mediated skin inflammation facilitates mast cell aggregation [61]. Therefore, the downregulation of Th17 signaling in skin lesions of ADSC-treated mice might partially explain observed decreases in mast cell aggregation relative to that in PBS-treated mice in the present study.

Although the mechanisms associated with the immunosuppressive effect of MSCs on Th17 cells have not been fully elucidated, several processes have been reported recently, including dependence on cell–cell contact-based inhibition and the production of soluble factors [62]. In the present study, ADSCs were found to exert a strong immunosuppressive effect on Th17 cells in co-culture assay. PD-L1 is expressed on the cytomembrane of MSCs and plays an important role in the negative regulation of immune responses [30, 63]. A previous study reported that co-culture of bone marrow-derived MSCs with Th17 lymphocytes led to a significantly increase in PD-L1 expression on the surface of MSCs, which inhibited the proliferation of Th17 cells, with the latter effect reversed by PD-L1-neutralizing antibodies [64]. The present study showed significant accumulation of PD-L1 during co-culture, indicating that inhibition of Th17 signaling by ADSCs might be partially attributable to cellular contact with Th17 cells via PD-L1. Additionally, MSC-derived PGE2 and TGF-β reportedly play a significant role in regulating multiple immune suppressive effects [31, 65, 66]. Upon exposure to Th17-skewing conditions, MSC-derived PGE2 inhibits IL-17A secretion through via cell contact-dependent mechanism [31, 67]. To assessed the immunoregulatory effects of secretory factors produced by ADSCs, Vasilev et al. [68] co-cultured ADSC-conditioned medium with peripheral blood mononuclear cells from rheumatoid arthritis patients, with the co-culture system demonstrating an increase in the Treg/Th17 ratio, as well as in the expression of TGF-β and a decrease in IL-17 production. In the present study, the co-culture ADSCs were found contained higher levels of PGE_2_ and TGF-β, relative to the monocultured cells, which might explain the immunosuppressive effect of ADSCs on Th17 cells. Collectively, the present study demonstrated that ADSCs exerted an immunosuppressive effect on Th17 cells, which might be attributable to both cell–cell contact inhibition and secretion of immunosuppressive cytokines; however, further study is required to clarify the exact mechanisms.

Therapeutic options for AD remain limited, in part because currently available models do not adequately capture all immune and barrier features of human AD skin [69–71], with OVA-induced and NC/Nga mice being one of the best animal models available [72]. However, there are diverse of immune responses reported between experimental AD studies, with these differences possibly resulting from the use of different animal models and methods [72–74]. Th17 signaling has been reported in various autoimmune disease. In particular, recent studies have suggested increased Th17 activation in Asian AD patients, where the percentage of Th17 signaling significantly correlated with AD severity [75–78]. Therefore, the present study provides some novel insights that broadens the overall understanding of and therapeutic approach to Th17 signaling in AD.

This study has some limitations. First, this study employed an OVA-induced AD mouse model. The use of more reliable animal models (i.e., xenografted or bioengineered skin-humanized mice) capable of capturing additional AD features might be desirable. Second, a recent study demonstrated that different MSC lines have different immunomodulatory properties [79]; therefore, a comparison of therapeutic effects between different MSC lines on AD mouse skin would deepen our understanding regarding the therapeutic mechanisms of AD treated with MSCs. Third, the therapeutic effects of stem cells are mainly attributable to their tissue regenerative capacity and immunomodulatory ability; however, in this study, only the immunomodulatory ability of ADSCs on AD was investigated. Further studies to clarify the tissue regenerative capacity of ADSCs on AD are required.

## Conclusions

MSC-based therapy is a promising and potent therapeutic approach to human allergic diseases, particularly AD. In this study, subcutaneous injection of ADSCs ameliorated AD by downregulating IL-17 secretion by Th17 cells in an OVA-induced mouse model. Additionally, this study employed RNA-sequencing technology to offer a comprehensive perspective on pathological changes in ADSC-treated AD skin. These findings can expand the knowledge concerning the mechanisms of MSC-based therapy for AD and offer insight into novel treatment strategies for allergic diseases using cell therapy. However, the systemic nature of AD indicates that systemic therapies are necessary to manage this disease. Therefore, further research should focus on developing multifaceted treatment strategies for AD, including both systemic and topical therapies. Furthermore, given the differences between animal models and humans, the therapeutic effect of ADSCs on human AD should be demonstrated via clinical studies in the future.

## Supplementary Information

**Additional file 1: Table S1.** Primer–probe sequences for the quantitative real-time polymerase chain reaction.

**Additional file 2: Fig. S1.** Multi-lineage differentiation of ADSCs. (a) Alcian Blue, (b) Alizarin Red, and (c) Oil Red O staining of ADSCs cultured in chondrogenic, osteogenic, and adipogenic differentiation mediums, respectively. Arrows indicate areas that were typically positive for each staining. Scale bar = 200 μm. ADSCs, Adipose-derived stem cells.

## Data Availability

The datasets used and/or analyzed during the current study are available from the corresponding author on reasonable request.

## Abbreviations

MSCs: Mesenchymal stem cells
AD: Atopic dermatitis
ADSCs: Adipose-derived stem cells
OVA: Ovalbumin
Th: Helper T
PBS: Phosphate-buffered saline
PBS+: PBS supplemented with 1% penicillin/streptomycin
SVF: Stromal vascular fraction
DMEM: Dulbecco’s modified Eagle’s medium
FBS: Fetal bovine serum
DMEM-low glucose+: DMEM-low glucose supplemented with 10% FBS and 1% penicillin/streptomycin
CO_2_: Carbon dioxide
NC: Normal control
COR: Cortisone
H&E: Hematoxylin and eosin
TB: Toluidine Blue
IgE: Immunoglobulin E
cDNA: Complementary DNA
PCR: Polymerase chain reaction
DEGs: Differentially expressed genes
FC: Fold change
padj: Adjusted *p* value
GO: Gene Ontology
KEGG: Kyoto Encyclopedia of Gene and Genome
RPMI: Roswell Park Memorial Institute
RPMI-1640+: RPMI-1640 supplemented with 10% FBS, 2 mM L-glutamine and 1% penicillin/streptomycin
qPCR: Quantitative real-time polymerase chain reaction
IL: Interleukin
IL-4R: IL-4 receptor
CCL20: C–C motif chemokine ligand 20
MMP12: Matrix metallopeptidase 12
IFN-γ: Interferon-gamma
TNF-α: Tumor necrosis factor-alpha
RORγT: RAR-related orphan receptor gamma T
PD-L1: Programed death ligand 1
TGF-β: Transforming growth factor-beta
PGE2: Prostaglandin E2
SD: Standard deviation
PCA: Principal component analysis
BP: Biological process
CC: Cellular component
MF: Molecular function
Treg: Regulatory T
