# Supplementary material for: Adipose-derived stem cells ameliorate atopic dermatitis by suppressing the IL-17 expression of Th17 cells in an ovalbumin-induced mouse model
Source: Stem Cell Res Ther. 2022 Mar 7;13:98. doi: 10.1186/s13287-022-02774-7 (PMC8900338; doi:10.1186/s13287-022-02774-7)
Supplement: Supplementary file 1 — Additional file 1: Table S1. Primer–probe sequences for the quantitative real-time polymerase chain reaction. [file 13287_2022_2774_MOESM1_ESM.docx]

Table S1. Primer-probe sequences for quantitative real-time polymerase chain reaction

| Sample source | Gene name | 5’-Forward-3’ | 5’-Reverse-3’ |
| --- | --- | --- | --- |
| Skin | Actb | GAGGTATCCTGACCCTGAAGTA | CACACGCAGCTCATTGTAGA |
|  | IL-13 | GCTGAGCAACATCACACAAG | AATCCAGGGCTACACAGAAC |
|  | IL-4 | GACGGCACAGAGCTATTGAT | GGATATGGCTCCTGGTACATTC |
|  | IL-4R | CCTACACTACAGGCTGATGTTC | TGGACCGGCCTATTCATTTC |
|  | IL-17A | CAAACATGAGTCCAGGGAGAG | GCTGAGCTTTGAGGGATGAT |
|  | CCL20 | CACAAGACAGATGGCCGATG | CAGCCCTTTTCACCCAGTTC |
|  | MMP12 | TTGACCCACTTCGCCAAAAG | GTGTGGAAATCAGCTTGGGG |
|  | IFN-γ | ATCGGCTGACCTAGAGAAGA | AGCCAAGATGCAGTGTGTAG |
|  | TNF-α | CTACCTTGTTGCCTCCTCTTT | GAGCAGAGGTTCAGTGATGTAG |
| Th17 cells | IL-17A | CAAACATGAGTCCAGGGAGAG | GCTGAGCTTTGAGGGATGAT |
|  | IL-17F | GTCGCCATTCAGCAAGAAATC | GAGCATCTTCTCCAACCTGAA |
|  | RORγt | CCTTCCCTCCACTCTATAAGGA | GTCAGAGGGCTGAAGGAAATAG |
| ADSCs | PD-L1 | GAGTGCAGATTCCCTGTAGAAC | CTCTCCTGCCACAAACTGAA |
|  | TGF-β | GGTGGTATACTGAGACACCTTG | CCCAAGGAAAGGTAGGTGATAG |
|  | PGE2 | CAGTCCAGTGCCAGTAAACA | CCCTTACACTTCTCCAATGAGG |
